# What the papers say

**DOI:** 10.1093/jhps/hnaa036

**Published:** 2020-10-26

**Authors:** Ali Bajwa

**Affiliations:** Consultant Orthopaedic Surgeon, Villar Bajwa Practice, The Princess Grace Hospital, London, UK

The *Journal of Hip Preservation Surgery* (*JHPS*) is not the only place where work in the field of hip preservation may be published. Although our aim is to offer the best of the best, we continue to be fascinated by work that finds its way into journals other than our own. There is much to learn from it so *JHPS* has selected six recent and topical subjects for those who seek a summary of what is taking place in our ever-fascinating world of hip preservation. What you see here are the mildly edited abstracts of the original articles, to give them what *JHPS* hopes is a more readable feel. If you are pushed for time, what follows should take you no more than 10 min to read. So here goes …

## THE ROLE OF THE ORTHOPAEDIC SURGEON IN THE COVID-19 ERA: CAUTIONS AND PERSPECTIVES

Ambrosio *et al*. [1] from the University of Rome, Italy report that the current coronavirus disease 2019 (COVID-19) pandemic has revolutionized global healthcare in an unprecedented way with considerable repercussions. Resource reallocation, socioeconomic confinement and reorganization of production activities are among the current challenges that are being faced both at the national and international level, in the backdrop of uncertainty and fear. Hospitals have been restructured to provide the best care for the COVID-19 patients while adopting the preventative strategies to avoid spreading the infection among healthcare providers and the patients affected by other diseases. As a consequence, the concepts of urgency and indications for elective treatment have been profoundly reshaped. The authors further state that several providers have been recruited to serve in the COVID-19 departments despite their differing original skills, resulting in a significant rearrangement of both the in-patient and outpatient care. Orthopaedic daily practices have been significantly affected by the pandemic. Surgical indications have been reformulated, with elective cases being promptly postponed and urgent interventions requiring exceptional attention, especially in suspected or COVID-19 patients. This has made a strong impact on the in-patient management, with the need for dedicated staff, patient isolation and restricted visiting-hour policies. On the other hand, the outpatient visits have been limited to reduce contact between patients and the hospital personnel, which has considerable consequences for post-operative quality of care and the human side of medical practice.

In this review, the authors aim to analyse the effects of the COVID-19 pandemic on orthopaedic practice. They state that particular attention will be dedicated to optimize surgical indications, perioperative care and safe management of both the inpatient and outpatient cohorts. They also consider the repercussions of the pandemic on orthopaedic residents’ training and, of course, the ethical implications.

## IDENTIFICATION OF KEY BIOMARKERS AND IMMUNE INFILTRATION IN THE SYNOVIAL TISSUE OF OSTEOARTHRITIS BY BIOINFORMATICS ANALYSIS

The authors from Wuhan University, Wuhan, China [2] report that osteoarthritis (OA) is the most common chronic degenerative joint disease and is mainly characterized by cartilage degeneration, subchondral bone hyperplasia, osteophyte formation and joint space reduction. Recent studies have shown that synovitis might also be an important pathological change of OA. However, the molecular mechanisms of synovitis in OA are still not well-understood.

The authors designed this study to identify key biomarkers and immune infiltration in the synovial tissue of OA by bioinformatics analysis.

The gene expression profiles of GSE12021, GSE55235 and GSE55457 were downloaded from the GEO database. The differentially expressed genes (DEGs) were identified by the LIMMA package in Bioconductor, and functional enrichment analyses were performed. A protein-protein interaction network (PPI) was constructed, and module analysis was performed using STRING and Cytoscape. The CIBERSORT algorithm was used to analyse the immune infiltration of synovial tissue between OA and normal controls.

The authors detected a total of 106 differentially expressed genes, including 68 down-regulated genes and 38 up-regulated genes. The PPI network was assessed, and the most significant module containing 14 hub genes was identified. Gene Ontology analysis revealed that the hub genes were significantly enriched in immune cell chemotaxis and cytokine activity. The KEGG pathway analysis showed that the hub genes were significantly enriched in the rheumatoid arthritis signalling pathway, IL-17 signalling pathway and cytokine–cytokine receptor interaction signalling pathway. The immune infiltration profiles varied significantly between OA and normal controls. Compared with normal tissue, OA synovial tissue contained a higher proportion of memory B cells, naive CD4+ T cells, regulatory T cells, resting dendritic cells and resting mast cells, while naive CD4+ T cells, activated NK cells, activated mast cells and eosinophils contributed to a relatively lower portion, which was significant. Finally, the expression levels of 11 hub genes were confirmed by RT-PCR.

The authors thus concluded that the hub genes and the difference in immune infiltration in synovial tissue between OA and normal controls might provide new insight for understanding OA development.

## MASSIVE CUTBACK IN ORTHOPAEDIC HEALTHCARE SERVICES DUE TO THE COVID-19 PANDEMIC

Liebensteiner *et al*. [3] report that this study was undertaken with the aim to investigate current possible cutbacks in orthopaedic healthcare due to the COVID-19 pandemic.

The study design was that of a structured survey. An online survey of the orthopaedic surgeons in the German-speaking Arthroscopy Society (Gesellschaft für Arthroskopie und Gelenkchirurgie, AGA) was performed. The survey consisted of 20 questions concerning four topics: 4 questions addressed the origin and surgical experience of the participant, 12 questions dealt with potential cutbacks in orthopaedic healthcare and the remaining four questions addressed the influence of the pandemic on the particular surgeon.

Of the 4234 contacted orthopaedic surgeons, 1399 responded. Regarding the arthroscopic procedures, between 10% and 30% of the participants stated that these were still being performed—with actual percentages depending on the specific joint and procedure. Only 6.2% of the participants stated that elective total joint arthroplasty was still being performed at their centre. In addition, physical rehabilitation and post-operative follow-ups were severely affected.

The authors noted that the orthopaedic healthcare services in Austria, Germany, and Switzerland are suffering a drastic cutback due to COVID-19. A dramatic reduction in arthroscopic procedures like rotator cuff repair and cruciate ligament reconstruction and an almost total shutdown of the elective total joint arthroplasty were reported. The authors felt that the long-term consequences cannot be predicted yet and that the described disruption in orthopaedic healthcare services has to be viewed as historic.

## RECOMMENDATIONS TO OPTIMIZE THE SAFETY OF ELECTIVE SURGICAL CARE WHILE LIMITING THE SPREAD OF COVID-19: PRIMUM NON-NOCERE

Gilat *et al*. [4] from Chicago, USA draw attention to the fact that COVID-19 has drastically altered our lives in an unprecedented manner, shuttering industries and leaving most of the country in isolation as we adapt to the evolving crisis. Orthopaedic surgery has not been spared from these effects, with the postponement of elective procedures in an attempt to mitigate disease transmission and preserve hospital resources as the pandemic continues to expand. During these turbulent times, the authors state, it is crucial to understand that although safety of patients and care providers is paramount, cancelling or postponing essential surgical care is not without consequences and may be irreversibly detrimental to patients’ health and quality of life in some cases. The optimal solution to how to balance effectively the resumption of standard surgical care while doing everything possible to limit the spread of COVID-19 is undetermined and could include such strategies as social distancing, screening forms and tests, temperature screening, segregation of in-patient and outpatient teams, proper use of protective gear, and the use of ambulatory surgery centres to provide elective, yet ultimately essential, surgical care while conserving resources and protecting the health of patients and healthcare providers. The authors emphasize that these recommendations do not and should not supersede the evolving guidelines from the United States Centers for Disease Control and Prevention, relevant federal, state and local public health organisations.

## THE COVID-19 OUTBREAK IN ITALY: PERSPECTIVES FROM AN ORTHOPAEDIC HOSPITAL

Grassi *et al.* [5] from the Rizzoli Institute, Bologna report that Italy is one of the most severely affected countries in the world by the recent COVID-19 outbreak. The aim of their report is to describe how COVID-19 affected the life and organization of one of the main orthopaedic hospitals of the country, and which measures were implemented to face the outbreak.

The study design involved personal interviews that were conducted with the four doctors involved in the management of COVID-19 outbreak in one of the main orthopaedic hospital of Italy.

The authors report that the hospital in question was reorganized, elective surgeries were cancelled, and only trauma surgeries were allowed, together with oncologic and urgent cases. Since the number of cases among patients and healthcare workers increased, the hospital management responded not only with a massive testing campaign aimed at detecting contact histories but also with an additional testing campaign for asymptomatic healthcare workers.

The main learning point, the authors conclude, is that any actions should be quick and decisive, as 1 week during the COVID-19 epidemic could make all the difference.

## TIME-SENSITIVE AMBULATORY ORTHOPAEDIC SOFT-TISSUE SURGERY PARADIGMS DURING THE COVID-19 PANDEMIC

The authors from Singapore [6] report that the timing of surgery for orthopaedic injuries continues to evolve, as an improved understanding of biology, healing and technological advances continues to challenge historical norms. With the growing COVID-19 pandemic that is stretching limited healthcare resources, postponing surgery becomes an inevitable and unenviable task for most orthopaedic surgeons. Hence a shift in outpatient paradigms is required to mitigate against poor patient outcomes.

A scoping review of five databases on surgical timing and orthopaedic soft-tissue injuries was performed. All randomized controlled trials, longitudinal cohort studies, retrospective case series, systematic reviews, meta-analyses and expert opinions were included for review, with 65 studies meeting the inclusion criteria.

Better outcomes appear to be associated with early surgery for subluxations (<1 week), recurrent dislocations (>2 episodes), ligamentous and tendinous injuries (<2 weeks) and bony avulsion injuries (<3 weeks). Spinal conditions with neurological compromise should be operated on within 24 h and spinal instability within 72 h to reduce the risk of complications and poor outcomes.

The authors conclude that most of the soft-tissue orthopaedic injuries can be managed with outpatient ambulatory surgery in a semi-elective setting. As the paradigm for outpatient surgery shifts due to technological advances and the COVID-19 pandemic, it is critical for surgeons to time their surgery appropriately to maintain the high standards of orthopaedic practice.

## CONFLICT OF INTEREST STATEMENT

None declared.
